# Analysis of cyanide exposure status in fire-related deaths using a physiologically based pharmacokinetic model

**DOI:** 10.1007/s11419-025-00713-8

**Published:** 2025-02-25

**Authors:** Kazuo Harada, Yuri Tokugawa, Kazunari Henmi, Yohei Miyashita, Yuji Sakahashi, Taichi Nishihori, Yukari Sakamoto, Chihpin Yang, Yu Isobe, Kana Sugimoto, Kentaro Nakama, Ryuichi Katada, Hiroshi Matsumoto

**Affiliations:** 1 Graduate School of Pharmaceutical Sciences, Osaka University, Yamadaoka 1-6, Suita, Osaka 565-0871 Japan; 2 School of Pharmaceutical Sciences, Osaka University, Yamadaoka 1-6, Suita, Osaka 565-0871 Japan; 3 Department of Legal Medicine, Graduate School of Medicine, Osaka University, Yamadaoka 2-2, Suita, Osaka 565-0871 Japan; 4 Education and Research Institute for Death Control and Prevention, Graduate School of Medicine, Osaka University, Yamadaoka 2-2, Suita, Osaka 565-0871 Japan; 5 Present Address: Osaka Rosai Hospital, Nagasonecho 1179-3, Kita-ku, Sakai, Osaka 591-8025 Japan; 6 Present Address: Department of Genomic Medicine, National Cerebral and Cardiovascular Center, Kishibe-Shimmachi 6-1, Suita, Osaka 564-8565 Japan; 7 Present Address: Omics Research Center, National Cerebral and Cardiovascular Center, Kishibe-Shimmachi 6-1, Suita, Osaka 564-8565 Japan; 8 Present Address: Planning and Personnel Welfare Division, Department of General Affairs, Osaka Prefectural Government, Otemae 2-Chome, Chuo-ku, Osaka, 540-8570 Japan; 9 Present Address: Division of Legal Medicine, Department of Community Medicine and Social Healthcare Science, Kobe University Graduate School of Medicine, 7-5-1, Kusunoki-cho, Chuo-ku, Kobe, Hyogo 650-0017 Japan

**Keywords:** Cyanide, Physiologically based pharmacokinetics, Fire-related death

## Abstract

**Purpose:**

Fire victims often inhale hydrogen cyanide (HCN) gas in addition to carbon monoxide. This study aimed to investigate the current prevalence of HCN inhalation among fire victims and assess the contribution of HCN as a toxic factor in fire-related deaths.

**Methods:**

The study included 29 cases of fire-related deaths, where autopsies were conducted at the Department of Legal Medicine, Osaka University, from April 2014 to March 2020. No resuscitation was performed before death was confirmed and blood samples were obtained from both the left and right cardiac chambers. Blood cyanide concentrations were measured. Additionally, a physiologically based pharmacokinetic model, as described by Stamyr et al. (Arch Toxicol 89:1287–1296, 2015), was used to simulate the time course of blood concentration changes for different inhaled HCN concentrations. The inhaled HCN concentration and inhalation time that minimized the difference between the measured and simulated blood concentrations were calculated.

**Results:**

Cyanide was detected in the cardiac blood of 76.3% of cases. In all instances, left cardiac blood concentrations were higher than those in the right cardiac blood. The simulations using the physiologically based pharmacokinetic model revealed eight cases where the inhaled HCN concentration exceeded 5000 ppm, with an inhalation time of less than 0.5 min.

**Conclusions:**

Many fire victims inhaled HCN gas, and in a few cases, it appears that death occurred rapidly after inhalation of high HCN concentrations. These findings suggest that the contribution of cyanide gas to fire-related deaths warrants closer examination.

**Supplementary Information:**

The online version contains supplementary material available at 10.1007/s11419-025-00713-8.

## Introduction

In 2022, there were approximately 36,000 fires in Japan, resulting in approximately 1400 deaths and 5700 injuries [1]. Causes of fire-related deaths include inhalation of combustion gases, oxygen deficiency, and burn injuries. The most common gases responsible for combustion poisoning are carbon monoxide (CO) and hydrogen cyanide (HCN) [2–4]. CO binds to hemoglobin in red blood cells, impairing oxygen transport [5], whereas cyanide (CN) binds to cytochrome c oxidase in the mitochondria, inhibiting cellular respiration [5, 6]. Both gases can lead to tissue oxygen deficiency. Severe CO poisoning occurs when blood carboxyhemoglobin levels (COHb%) exceed 50%, and levels over 70% are often fatal [7]. Similarly, severe CN poisoning is associated with blood concentrations above 1 µg/mL, with levels over 3 μg/mL being potentially fatal [8]. CO is generated by incomplete combustion and is almost always present in fires, making blood COHb% a routine test in forensic autopsies of fire-related deaths. CN is produced from the combustion of nitrogen-containing polymers [2–4]. Although it is less common than CO, fire victims still frequently inhale CN [9–15]. However, fewer institutions measure CN levels compared to COHb%, increasing the risk of underestimating its contribution to fire-related fatalities.

Furthermore, an experiment using rabbits by Kagehara showed that low concentrations of inhaled HCN resulted in a dose-dependent increase in blood CN levels. However, when high concentrations of HCN were inhaled, leading to respiratory arrest within minutes, blood CN levels did not increase proportionally to the inhaled dose and were lower than those of increases involving lower HCN concentrations [16]. Although the molecular mechanism behind this observation remains unclear, the study suggests that diagnosing HCN poisoning based solely on postmortem blood CN levels may be unreliable. This highlights the need for new diagnostic methods to assess HCN gas poisoning accurately.

In this study, we aimed to investigate the current extent of HCN inhalation among fire victims and assess its contribution as a toxic agent in fire-related deaths. To achieve this objective, we measured CN concentrations in left and right cardiac blood samples from 29 cases of fire-related deaths. Additionally, we used the physiologically based pharmacokinetic (PBPK) model developed by Stamyr et al. [17] to simulate the time course of blood concentration changes for different inhaled HCN concentrations. We estimated the inhaled HCN concentration and inhalation time by minimizing the difference between the measured and simulated left and right cardiac blood concentrations.

## Materials and methods

### Cases

The study included 29 cases of fire-related deaths, with autopsies conducted at the Department of Legal Medicine, Osaka University, between April 2014 and March 2020. In these cases, no resuscitation was performed before death was confirmed and blood samples were collected from both the left and right cardiac chambers. The following measurements were conducted at the Department of Legal Medicine.

### Reagents

Cyanoline blue, pyridine, and sodium thiocyanate were purchased from FUJIFILM Wako Pure Chemical Corporation (Osaka, Japan). Phosphoric acid, dihydrogen phosphate dihydrate, ethyl acetate, sodium tetraborate decahydrate, and sodium hydroxide were acquired from Kishida Chemical Co., Ltd. (Osaka, Japan). A standard solution for cyanide was purchased from Kanto Chemical Co., Inc. (Tokyo, Japan). Sodium *p*-toluenesulfonchloramide trihydrate, pentafluorobenzyl bromide (PFB-Br), and benzyldimethyltetradecylammonium chloride (TDMBA) were purchased from Tokyo Chemical Industry Co., Ltd. (Tokyo, Japan). Chloramine T solution was prepared by dissolving 20 mg of sodium *p*-toluenesulfonchloramide trihydrate in 10 mL of pure water, followed by mixing 5 mL of this solution with 15 mL of a 1 mol/L phosphate solution. The pyridine-pyrazolone reagent was made by dissolving 0.27 g of cyanoline blue in 20 mL of pyridine, and then adding 100 mL of pure water. The standard solution for the CN calibration curve was prepared by diluting a CN standard solution with 0.1 mol/L sodium hydroxide (NaOH) solution. For the thiocyanate (SCN) calibration curve, 13.9 mg of sodium thiocyanate was dissolved in 0.1 mol/L NaOH solution, brought to a final volume of 10 mL to create the stock solution, and then diluted with 0.1 mol/L NaOH solution.

### Carboxyhemoglobin percentage measurement

COHb% was measured immediately after blood sampling during the forensic autopsy using a blood gas analyzer GASTAT-700 (Techno Medica Co., Ltd., Kanagawa, Japan).

### Blood cyanide concentration measurement

The measurements were performed using the method described by Okada and Miyaguchi [18]. Blood samples, which had been frozen and stored at -30 °C after collection, were used. A 0.5 mL genetic analyzer sample tube (Applied Biosystems, Waltham, MA, USA) was inserted into a 5 mL self-standing tube (Ina-optika Corporation, Osaka, Japan) or a 5 mL screw-cap tube (SARSTEDT AG & Co., KG, Nümbrecht, Germany), which served as a disposable microdiffusion device. A total of 400 μL of 0.1 mol/L NaOH solution was added to the genetic analyzer sample tube, while 200 μL of blood, diluted two-fold with ultrapure water, and 100 μL of 10% phosphoric acid were added to the outer chamber. The cap was then sealed and gently mixed. The tube was placed on a shaker, stirring at 170 rpm at 25 °C for 2 h. From the disposable microdiffusion device, 50 μL of the CN standard solution for the calibration curve and the NaOH solution were transferred to a 96-well plate and cooled on ice. To ensure reproducibility, two wells were prepared for each sample. Next, 10 μL of freshly prepared chloramine T reagent was added to each well and cooled on ice for 2 min. Then, 150 μL of pyridine-pyrazolone reagent was added, and the plate was left at room temperature in the dark for 40 min. The absorbance at 630 nm was then measured using an SH-9000 Lab microplate reader (Hitachi High-Tech Co., Tokyo, Japan). The amount of SCN converted to CN during this method was below the detection limit (0.2 μg/mL) and did not affect the quantitative values. The lower limit of CN quantification in blood was 0.4 μg/mL in our experiment.

### Blood thiocyanate concentration measurement

The measurements were performed using a modified version of the method described by Kage et al. [19]. Whole blood stored at − 30 °C was used. A 100-μL blood sample was transferred into a 1.5-mL microtube, to which 125 μL of 20 mM PFB-Br in ethyl acetate and 200 μL of 5 mmol/L TDMBA in saturated Na_2_B_4_O_7_ solution were added. The mixture was vigorously vortexed for 1 min, heated at 55 °C for 30 min, and then centrifuged at 10,000×*g* for 3 min. A 100-μL portion of the supernatant was then analyzed using gas chromatography/mass spectrometry (GC/MS). GC/MS analysis used a GCMS-QP2010 Ultra system with an AOC-20i and AOC-20 autosampler (Shimadzu, Kyoto, Japan). The column was a DB-5 ms (0.25 mm i.d. × 30 m, 0.25 μm thickness, Agilent Technologies, CA, USA). Helium was used as the carrier gas, and the flow rate was adjusted to maintain a column linear velocity of 30.4 cm/s. The sample injection volume was 1 μL with splitless mode. The vaporization chamber temperature was set to 200 °C. The column oven temperature was held at 50 °C for 3 min, then increased to 250 °C at a rate of 20 °C/min, and maintained at 250 °C for 5 min. Sample ionization was conducted using the electron ionization (EI) method. The ion source temperature was set at 230 °C, and the interface temperature was 280 °C. The detector voltage was activated 3 min after sample injection, and data acquisition continued for 18 min. Selected ion monitoring (SIM) was performed for target ions at *m/z* 157, 161, 181, 188, 207, and 239, with an event time of 0.30 s per ion.

### Simulation of blood cyanide concentration during HCN gas inhalation

The PBPK model and parameters used in the simulation were based on those proposed by Stamyr et al. [17]. This model consists of a set of simultaneous ordinary differential equations, with the CN concentration in each compartment as a variable, and was numerically solved using the Runge–Kutta method. Simulations were conducted using Python 3.8.3, with additional libraries such as numpy and scipy installed. The script is provided in Supplemental Material 1. The following method was used to estimate the inhaled HCN gas concentration and inhalation time: The inhaled HCN gas concentration was set, and CN concentrations in arterial and venous blood were calculated at 0.05-min intervals over an inhalation period ranging from 0 to 30 min. The difference between the measured CN concentration in the right heart blood and the simulated venous blood CN concentration and between the measured CN concentration in the left heart blood and the simulated arterial blood CN concentration were calculated. The sum of the squares of these differences was then computed. This calculation was repeated for inhaled HCN gas concentrations ranging from 0 to 18,000 ppm, with increments of 12 ppm. The inhaled HCN concentration and inhalation time that minimized the sum of squares were estimated as the values for that particular case (see Supplemental Material 2). These calculations were performed using Python, with the relevant scripts provided in Supplemental Material 3.

## Results

### Blood cyanide concentrations in fire-related death cases

**Fig. 1:**
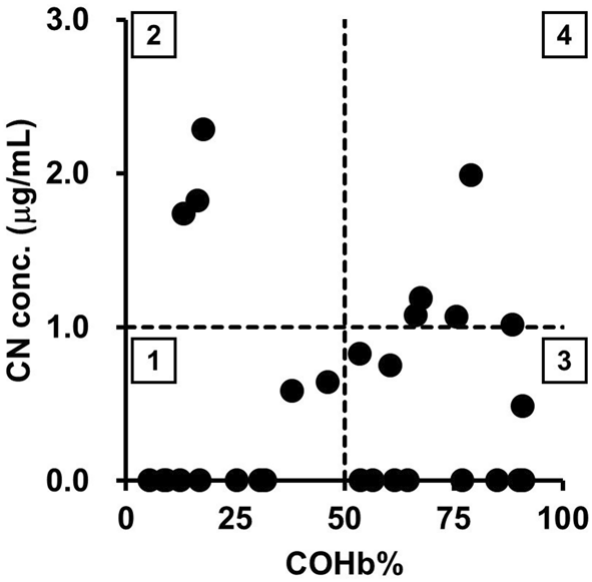
Scatter plot of right cardiac blood carboxyhemoglobin percentage (COHb%) and cyanide (CN) concentration in the cases analyzed in this study. The dashed lines represent the thresholds for COHb% and CN concentration considered as criteria for poisoning per references 15 and 16. The numbers in the boxes indicate the following zones: zone 1: individuals with sub-lethal levels of COHb% and CN in the blood, zone 2: individuals whose death was primarily caused by HCN, zone 3: individuals whose death was primarily caused by CO, zone 4: cases where both gases could have contributed to the cause of death

The CN and SCN concentrations in the left and right cardiac blood of the cases were measured. Table 1 provides these results, along with the age and sex of each case, and the COHb% levels in the left and right cardiac blood from blood gas tests conducted during the autopsy. In non-smoking, healthy individuals, COHb% is typically below 2.0% [20], but in all cases of this study, COHb% levels exceeded this threshold. CN was detected in 23 of the cases (79.3%) in the left or right cardiac blood, with a detection limit of 0.2 µg/mL for CN in this study. SCN was detected in all cases, with concentrations ranging from 0.92 to 8.5 μg/mL. In poisoning diagnosis, venous blood results are commonly used. Ferrari et al. proposed a method for assessing the severity of poisoning by setting thresholds for COHb% at 50% and CN at 1 μg/mL [13, 14]. Based on this method, the results in Table 1 were visualized in Fig. 1, with the right cardiac blood serving as a proxy for venous blood.

**Table 1:** Age, sex, cyanide concentrations, carboxyhemoglobin%, and thiocyanate concentrations in the left and right cardiac blood of cases analyzed in this study

No.	Age	Sex	CN conc. (μg/mL)		COHb%		SCN conc. (μg/mL)	
			R	L	R	L	R	L
1	89	F	2.0	3.7	79	94	2.2	2.1
2	78	M	1.7	2.9	13	14	1.7	1.4
3	69	F	ND	Trace	65	77	3.1	3.6
4	73	F	0.64	0.67	46	46	2.0	2.1
5	70	M	0.58	2.7	38	42	2.6	1.5
6	56	F	Trace	0.80	57	49	2.8	2.5
7	65	M	1.2	1.7	68	69	3.2	2.8
8	60	M	ND	ND	9.2	10	5.3	5.0
9	79	M	1.1	11	66	72	4.5	2.4
10	71	M	2.3	4.0	18	18	2.8	2.3
11	71	M	Trace	0.86	91	96	1.6	1.3
12	69	M	ND	1.4	54	54	2.4	1.6
13	78	M	0.75	4.0	61	76	3.0	2.0
14	55	F	ND	0.43	17	19	3.1	2.6
15	32	F	ND	Trace	62	44	8.5	7.9
16	32	F	ND	ND	26	25	3.8	3.7
17	80	M	0.48	2.7	91	93	2.5	1.5
18	82	M	1.0	3.1	89	91	4.1	2.3
19	78	F	1.1	2.4	76	82	2.7	1.8
20	70	M	ND	ND	5.4	6.1	0.98	1.0
21	81	F	ND	0.48	31	37	2.0	-
22	79	F	ND	Trace	32	32	1.6	1.3
23	77	M	0.82	1.8	54	54	3.0	2.7
24	54	M	ND	ND	90	100	0.94	0.92
25	58	M	1.8	2.9	16	9.7	3.8	4.3
26	60	M	ND	ND	8.7	6.1	2.6	2.5
27	49	M	Trace	Trace	13	13	6.4	6.5
28	39	M	Trace	Trace	77	77	7.9	7.6
29	31	F	ND	ND	85	84	2.2	1.5

Detection limit and lower limit of quantification are 0.2 and 0.4 μg/mL, respectively

*F* female, *M* male, *R* right cardiac blood, *L* left cardiac blood, *ND* not detected, – no data due to shortage of sample

**Fig. 2:**
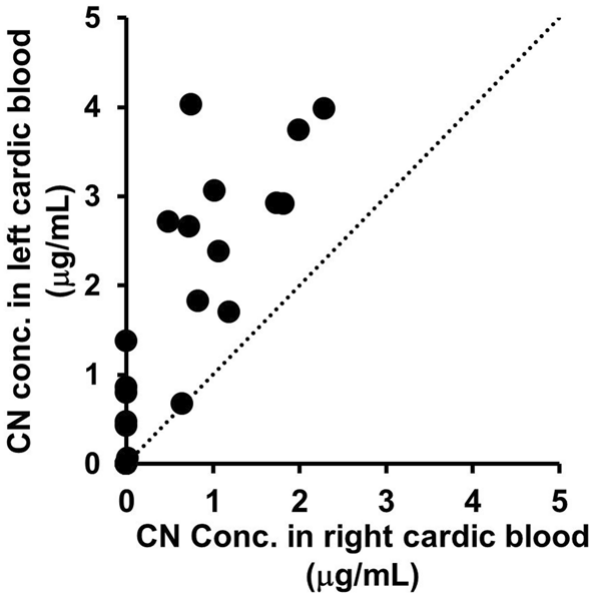
Scatter plot of cyanide (CN) concentrations in the left and right cardiac blood in the cases analyzed in this study. The dashed line indicates equal concentrations of CN in the left and right cardiac blood

A scatter plot comparing CN concentrations in the left and right cardiac blood is shown in Fig. 2. In all cases, the left cardiac blood displayed higher CN levels than those in the right cardiac blood.

### Simulation of time-course changes in blood cyanide concentration during hydrogen cyanide gas inhalation

**Fig. 3:**
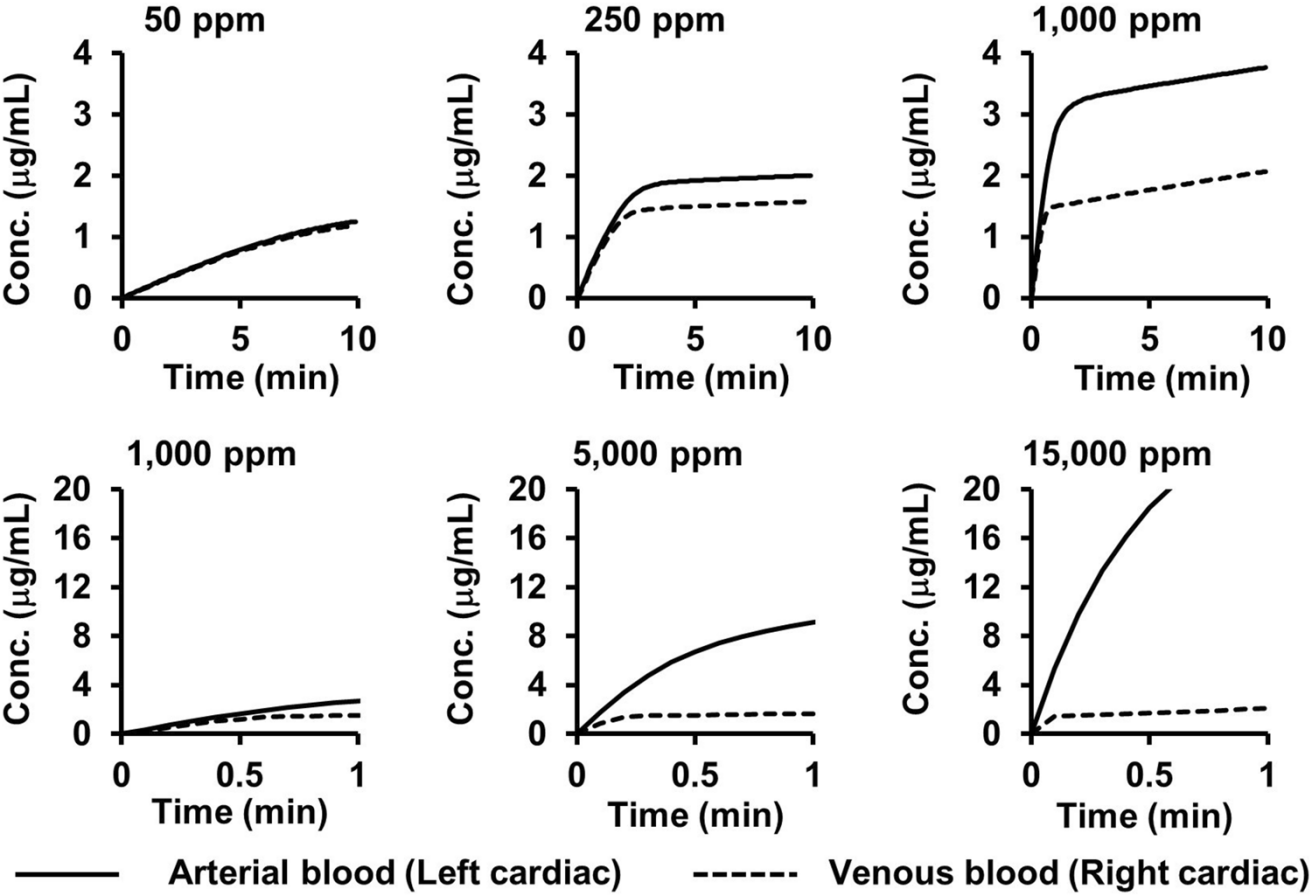
Simulation results of cyanide (CN) concentrations in the left and right cardiac blood when hydrogen cyanide gas is inhaled. The set value of the inhaled hydrogen cyanide gas concentration is indicated at the top of each graph. The top three graphs show the time-course changes in CN concentrations from the start of inhalation up to 10 min. The bottom three graphs show the time-course changes in CN concentrations from the start of inhalation up to 1 min

Using the PBPK model for HCN gas inhalation described by Stamyr et al. [17], we simulated the time-course changes in CN concentration in arterial and venous blood. The simulation was programmed in Python, with the script provided in Supplemental Material 1. Figure 3 illustrates the simulation results for inhaled HCN gas concentrations of 50, 250, 1000, 5000, and 15,000 ppm. Following the onset of inhalation, CN concentrations in both arterial and venous blood increased rapidly before the rate of increase gradually slowed. This deceleration occurred slightly earlier in venous blood, leading to a concentration difference between arterial and venous blood that continued to widen. Shortly thereafter, the rate of increase in arterial blood decelerated. Even after the slowdown in arterial and venous concentrations, the difference between them continued to expand slightly.

### Estimation of inhaled hydrogen cyanide gas concentration and inhalation time in fire-related death cases

Based on the simulation results, we estimated the inhaled HCN gas concentration and inhalation duration for cases where blood CN concentration values were available. PBPK simulations were conducted for inhaled HCN concentrations ranging from 0 to 18,000 ppm at 12-ppm intervals and for inhalation times from 0 to 30 min at 0.05-min intervals. We calculated the differences between the simulated CN concentrations in arterial blood and the measured CN concentrations in left cardiac blood and between venous blood and right cardiac blood for all inhalation concentrations and times. The inhalation concentration and time that minimized the sum of squared differences were selected as the most probable values. A conceptual diagram and Python script for this calculation are available in Supplemental Materials 2 and 3, respectively. The estimated inhaled HCN concentrations and inhalation times are represented in Table 2. For high inhaled gas concentrations (≥ 5000 ppm), the estimated inhalation times were very short, ranging from 0.05 to 0.4 min. In contrast, for concentrations below 5000 ppm, the estimated inhalation times exceeded 1 min.

**Table 2:** Inhaled hydrogen cyanide gas concentration and time estimated from the left and right cardiac blood measurements and physiologically based pharmacokinetic simulations

No	CN conc. (μg/mL)		SCN conc. (μg/mL)		Estimated inhaled HCN gas conc	Estimated inhalation time
	R	L	R	L	(ppm)	(min)
High-concentration, short-term inhalation (Ca_high_T_short_) group						
5	0.58	2.7	2.6	1.5	14,100	0.05
7	1.2	1.7	3.2	2.8	1260	0.40
9	1.1	11	4.5	2.4	16,212	0.20
13	0.75	4.0	3.0	2.0	11,040	0.10
17	0.48	2.7	2.5	1.5	14,280	0.05
18	1.0	3.1	4.1	2.3	16,632	0.05
19	1.1	2.4	2.7	1.8	6624	0.10
23	0.82	1.8	3.0	2.7	5004	0.10
Low-concentration, long-term inhalation (Ca_low_T_long_) group						
1	2.0	3.7	2.2	2.1	1032	8.35
2	1.7	2.9	1.7	1.4	696	6.60
4	0.64	0.67	2.0	2.1	84	2.40
10	2.3	4.0	2.8	2.3	996	13.65
25	1.8	2.9	3.8	4.3	648	9.10

Case No., CN, and SCN concentrations are the same as those in Table 1

*R* right cardiac blood, *L* left cardiac blood

### Relationship between estimated inhalation time and thiocyanate concentration in the left and right cardiac blood

**Fig. 4:**
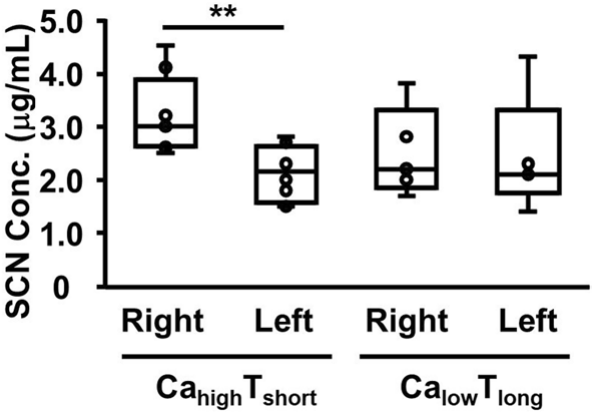
Comparison of distributions of thiocyanide concentrations in the left and right cardiac blood grouped per estimated inhaled hydrogen cyanide concentration and time. Cases were categorized into the low-concentration (less than 5000 ppm), long-term (1 min or more) inhalation (Ca_low_T_long_) group and high-concentration (5000 ppm or more), short-term (less than 1 min) inhalation (Ca_high_T_short_) group. An asterisk indicates that the difference was significant by *T*-test at the 1% significance level

Based on the estimated inhaled gas concentration and time, the cases were categorized into the low-concentration (less than 5000 ppm), long-term (1 min or more) inhalation (Ca_low_T_long_) group and the high-concentration (5000 ppm or more), short-term (less than 1 min) inhalation (Ca_high_T_short_) group. SCN concentrations in the left and right cardiac blood were compared between these groups. In the Ca_low_T_long_ group, no significant differences were observed between SCN concentrations in the left and right cardiac blood. However, in the Ca_high_T_short_ group, the right cardiac blood SCN concentration was significantly higher than that of the left cardiac blood (*p* = 0.0038, *T*-test) (Fig. 4).

## Discussion

Although it is well known that HCN gas is often inhaled in fire-related deaths, the number of facilities conducting forensic autopsy tests for HCN remains limited. In this study, we measured CN concentrations in left and right cardiac blood samples from 29 fire-related death cases. Our results showed that 79% of cases had CN concentrations above the detection limit of 0.2 µg/mL in either the left or right cardiac blood (Table 1). In all cases where CN was detected, the left cardiac blood had higher CN concentrations than the right cardiac blood (Table 1, Fig. 2), indicating that HCN was inhaled as a gas. This suggests that HCN gas was generated at many fire scenes and was inhaled by individuals present at those scenes.

Furthermore, poisoning was diagnosed based on COHb% and CN concentrations in the right cardiac blood, which can represent venous blood (Fig. 1). Our analysis identified three cases where the COHb% was below 50%, but the CN concentration exceeded 1 µg/mL (Zone 2). Additionally, there were two cases (Nos. 4 and 5 in Table 1) where COHb% and CN concentrations did not reach toxic thresholds (Zone 1) but were close to 50% and 1 µg/mL, respectively. This suggests that death in these cases may have resulted from the combined effects of both gases. Based on these findings, it is clear that measuring blood CN concentrations is crucial in fire-related death investigations.

However, a study with rabbits demonstrated that when high concentrations of inhaled CN caused immobilization and death, the CN concentration in the blood did not correlate with the inhaled levels and was lower compared to cases involving lower CN inhalation concentrations [16]. Similarly, Purser et al., using monkeys, showed that the concentration of inhaled HCN gas does not necessarily correspond with the CN levels in the blood [21]. Thus, relying solely on CN concentrations in venous blood poses a risk of misdiagnosis, a concern raised by Htike et al. [15]. The mechanism behind the lack of correlation between blood CN concentrations and high inhaled HCN levels remains unclear. We speculate that high concentrations of CN expelled from the left ventricle may activate chemoreceptors, stimulating the respiratory center and causing respiratory arrest [5, 6, 22]. If respiratory arrest occurs rapidly, death may happen before CN concentrations in venous blood can rise. Given these findings, we explored whether it might be possible to derive more accurate information on HCN inhalation by analyzing both venous and arterial blood CN concentrations. Using the PBPK model developed by Stamyr et al., we simulated the time course of blood CN concentrations during HCN gas inhalation. The goal was to calculate the inhaled HCN gas concentration and inhalation time to match the measured CN levels in the left and right cardiac blood. We achieved this by minimizing the sum of squares of the difference between the simulated results and the measured CN concentrations. This scenario was inferred to represent the conditions experienced by the deceased at the fire scene. The findings are detailed in Table 2. From the results, we classified the subjects into those with an inhaled CN gas concentration below 5000 ppm and an inhalation time of 1 min or more (Ca_low_T_long_) and those with an inhaled CN gas concentration of 5000 ppm or higher and an inhalation time of less than 1 min (Ca_high_T_short_). Inhaled HCN gas concentrations above 2000 ppm are known to cause instant death [23], and while the Ca_low_T_long_ group involved lower concentrations, the Ca_high_T_short_ group likely experienced immediate death after inhaling HCN gas. This suggests that the individuals in the Ca_high_T_short_ group may have stopped breathing almost instantly after exposure to high-concentration HCN at the fire scene. In cases classified as part of the Ca_high_T_short_ group, cases 5, 13, and 23 did not exhibit right cardiac blood CN concentrations above 1 µg/mL, which is considered within the toxic range. This finding highlights the potential risk of misdiagnosis when relying solely on CN concentrations in venous blood.

When cases were divided into the Ca_high_T_short_ and Ca_low_T_long_ groups, and SCN concentrations in the left and right cardiac blood were compared, a significant difference was observed in the Ca_high_T_short_ group: the right cardiac blood concentration was higher than the left. No such difference was observed in the Ca_low_T_long_ group. Although the exact mechanism behind this phenomenon remains unclear, it suggests that in the Ca_high_T_short_ group, a substantial amount of HCN gas was inhaled. This likely led to the partial metabolism of HCN into SCN, resulting in a significant increase in SCN concentrations in the venous blood.

A limitation of this study is that the accuracy of the simulation has not been fully validated. In our experiment, the uncertainty in CN measurements was approximately ± 15%. To assess the impact of this uncertainty, cases No. 7, 10, and 17 were re-evaluated by applying a ± 15% deviation to the measured CN values in the left and right cardiac blood. The results are provided in Supplemental Material 4. For case No. 17, the inhalation concentration ranged from 11,988 to 16,632 ppm, with an inhalation time of 0.05 min, suggesting a consistent estimate. For case No. 10, the inhalation concentration varied significantly from 588 to 1560 ppm, with inhalation times between 5.10 and 29.90 min, yet it still fits the criteria for the Ca_low_T_long_ group. Case No. 7 could mostly be distinguished from the Ca_high_T_short_ group; however, when the right and left cardiac CN concentrations were 1.380 and 1.955 µg/mL, respectively, it was interpreted as part of the Ca_low_T_long_ group, indicating a potential discrepancy. To enhance the reliability of the simulation, minimizing the measurement uncertainty of CN is essential. Additionally, the parameters used in this study were based on the values provided by Stamyr et al. [17]. However, these may require adjustments depending on individual differences in corpses. Variables such as body weight, body fat percentage, and lung capacity significantly influence CN distribution and inhalation volume, which can impact the simulation results. Refining the accuracy of these parameters is necessary. Moreover, the study assumed a constant HCN gas concentration during inhalation, which is unlikely in real fire scenarios where concentrations fluctuate dramatically over short periods. Therefore, future studies should validate how accurately the simulation represents real-life conditions.

Furthermore, a limitation of this study is that it did not account for the effects of methemoglobin, which is generated by nitrogen oxides present in combustion gases and binds to CN in the blood to detoxify it [15]. The PBPK model used in this study included CN metabolism; however, it did not clearly define the contribution of methemoglobin. The most significant finding of this study is that even when CN concentrations in venous blood are low, cases of respiratory arrest and death may occur after inhaling high concentrations of HCN gas over a short period. In such cases, we speculate that there may not have been sufficient time for methemoglobin to be produced, minimizing its impact on death. However, this remains speculative, and we believe that the role of methemoglobin should be considered in future research.

Another important aspect to consider is the potential influence of postmortem redistribution on the observed differences in CN concentration between left and right cardiac blood. CN is known to accumulate in high concentrations in the lungs [15]. It is highly likely that CN accumulated in the lungs from the beginning of the fire until death diffused into the left atrium through postmortem distribution, resulting in an increased CN concentration in the left cardiac blood. However, if the postmortem CN concentration measured in left cardiac blood is considered to represent the combined CN concentrations in both lung tissue and left cardiac blood immediately before death, and if the arterial blood compartment in the PBPK model is assumed to represent a combination of the lung and arterial blood, then the current PBPK model may already account for the effects of postmortem changes. We plan to conduct retrospective observational studies using autopsy records and animal experiments to investigate the effects of postmortem CN redistribution and incorporate these findings into the PBPK model. However, as this is challenging to achieve in a short timeframe, we intend to pursue this research in the future.

Despite potential accuracy limitations, analyzing the venous and arterial blood rather than focusing solely on the venous blood provides valuable insights. By examining the balance of CN concentrations between the two, it is possible to infer the circumstances of HCN inhalation better, yielding crucial information for forensic examinations. We emphasize that accurately estimating the circumstance of death in fire-related fatalities contributes to a precise diagnosis of the cause of death. Diagnosing CN poisoning based solely on CN concentrations in venous blood poses a potential risk. Using the PBPK model, if a victim is classified into the Ca_high_T_short_ group, it allows us to identify the possibility of death due to CN poisoning, even when venous blood CN concentrations are low. Moreover, in forensic autopsies, it is important not only to determine the cause of death but also to understand the circumstances of fire. This includes identifying what materials were burning and whether the fire had the potential for significant toxic gas production. Clarifying these factors makes it possible to assign responsibility for the fire and aids in developing measures to prevent fire-related fatalities. For example, if many cases are grouped as Ca_high_T_short_ deaths, preventive measures should prioritize the use of furniture and building materials that do not generate HCN gas and the development of HCN gas removal methods using alkaline agent. Conversely, if many cases are grouped as Ca_low_T_long_ deaths, ensuring proper evacuation routes and guidance during emergencies would take precedence as preventive strategies.

## Conclusions

In this study, blood CN concentrations were analyzed for 29 fire-related deaths. CN was detected in a significant proportion of cases (79%). The measurements of CN concentrations in both left and right cardiac blood were performed, and a PBPK model was employed to simulate blood CN concentrations during HCN gas inhalation. This approach allowed for estimating inhaled gas concentrations and inhalation times for each case. The findings suggested that a few individuals died rapidly due to inhalation of high-concentration gas, whereas others were exposed to lower concentrations over an extended period. However, several limitations remain. These include the inability to clearly account for the effects of methemoglobin and postmortem distribution in the PBPK model, the inability to verify the influence of fluctuations in parameters such as body weight and body fat percentage of the deceased, and concerns regarding the accuracy of the simulation. Despite these challenges measuring CN concentrations in the left and right cardiac blood samples and utilizing a PBPK model for HCN inhalation proves to be a valuable method for estimating the circumstances of death in fire-related fatalities.

## Supplementary Information

Below is the link to the electronic supplementary material.

Supplementary file1 (DOCX 138 KB)

## Data Availability

The datasets generated during the current study are available from the corresponding author upon reasonable request.
