# Supplementary material for: Analysis of cyanide exposure status in fire-related deaths using a physiologically based pharmacokinetic model
Source: Forensic Toxicol. 2025 Feb 25;43(2):247–55. doi: 10.1007/s11419-025-00713-8 (PMC12241193; doi:10.1007/s11419-025-00713-8)
Supplement: Supplementary file 1 — Supplementary file1 (DOCX 138 KB) [file 11419_2025_713_MOESM1_ESM.docx]

**Supplemental materials**

**Analysis of cyanide exposure status in fire-related deaths using a physiologically-based pharmacokinetic model**

Kazuo Harada, Yuri Tokugawa, Kazunari Henmi, Yohei Miyashita, Yuji Sakahashi, Taichi Nishihori, Yukari Sakamoto, Chihpin Yang, Yu Isobe, Kana Sugimoto, Kentaro Nakama, Ryuichi Katada, Hiroshi Matsumoto

**Supplemental material 1** Python script for blood cyanide concentration simulation

**Supplemental material 2** Conceptual image of the method for estimating inhaled hydrogen cyanide gas concentration and time

**Supplemental material 3** Python script for estimating inhaled hydrogen cyanide gas concentration and time

**Supplemental material 4** Sensitivity analysis results for inhaled hydrogen cyanide gas concentration and time

**Supplemental material 1 Python script for blood cyanide concentration simulation**

Note: By entering the inhaled cyanide gas concentration you want to simulate after “Ca=” in the following script and executing it, a graph showing the time-course changes of cyanide concentrations in the arterial (left cardiac) and venous (right cardiac) blood can be obtained.

# -*- coding: utf-8 -*-

# preparation of numpy and plot

import numpy as np

from scipy.integrate import solve_ivp

import matplotlib.pyplot as plt

# definition of parameters

Ppa = 281 # Ppa: Partition coefficients of plasma/air

Php = 5.1 # Php: Partition coefficients of liver/plasma

Pmp = 2.8 # Pmp: Partition coefficients of muscle/plasma

Pop = 5.4 # Pop: Partition coefficients of other tissues/plasma

Qalv = 16.5 # Qalv: Alveolar ventilation

Qtot = 10.7 # Qtot: Cardiac output

Qh = 1.6 # Qh: Liver blood flow

Qm = 4.9 # Qm: Muscle blood flow

Qo = 4.2 # Qo: Other tissues blood flow

Fp = 0.6 # Fp: Plasma fraction in whole blood

Cle = 65.9 # Cle: Clearance flow between blood and erythrocytes

Vp = 2.9 # Vp: Compartment volume of plasma

Ve = 1.9 # Ve: Compartment volume of erythrocytes

Vh = 1.6 # Vh: Compartment volume of liver

Vm = 35 # Vm: Compartment volume of muscle

Vo = 18 # Vo: Compartment volume of other tissues

Vb = 4.8 # Vb: Compartment volume in whole blood

Emax = 140 # Emax: Maximum binding to erythrocytes

Kaff = 1 # Kaff: Affinity constant to erythrocytes from plasma

Kscn = 0.01 #Kscn : second-order rate constant for thiocyanate formation

Kfs = 2.2 # Kfs: zero-order formation rate of sulfur donors

Kes = 0.0027 # Kes: first-order elimination rate constant

Clr = 0.0041 # Clr: first-order metabolic clearance of CN via remaining pathways

Ca = # CN conc. in air (umol/L), 1 umol/L = 27 ug/L = 24 ppm, 270 ppm = 0.30 mg/L = 11 umol/L

# definition of variables

# C = [0 Cp, 1 Ce, 2 Ch, 3 S, 4 Cm, 5 Co]

init = [0, 0, 0, 1, 0, 0]

#Cp: CN conc. in plasma

#Ce: CN conc. in erythrocytes

#Ch: CN conc. in liver

#S: sulfur donors conc.

#Cm: CN conc. in muscle

#Co: CN conc. in other tissues

# Setting of time span of simulation

t_span = [0.0, 10.0]

dt = 0.1

t_eval = np.arange(*t_span, dt)

# Definition of ordinary differential equation and initial values

def func(t,C):

Cp, Ce, Ch, S, Cm, Co = C

dCp = (Qalv*(Ca-(Cp/Ppa))+Cle*(Ce/(Emax/(Cp+Kaff))-Cp)+Fp*Qtot*(((Qh*Ch)/Php+(Qm*Cm)/Pmp+(Qo*Co)/Pop)/Qtot-Cp))/Vp

dCe = Cle*(Cp-(Ce/(Emax/(Cp+Kaff))))/Ve

dCh = (Fp*Qh*(Cp-(Ch/Php))-Kscn*Ch*S-(Kfs-Kscn*Ch*S-Kes*S))/Vh

dS = Kfs-Kscn*Ch*S-Kes*S

dCm = Fp*Qm*(Cp-Cm/Pmp)/Vm

dCo = Fp*Qo*(Cp-Co/Pop)/Vo

return np.array([dCp, dCe, dCh, dS, dCm, dCo])

sol = solve_ivp(func, t_span, init, method='RK45', t_eval=t_eval)

Cp, Ce, Ch, S, Cm, Co = sol.y

#Metscn: CN conc. metabolized via the SCN route

#Cb: CN conc. in whole blood

#Cv: CN conc. in vein (mixed venous plasma)

#Cvb: CN conc. in whole blood in vein

Metscn = Kscn*Ch*S

Cb = (Vp*Cp+Ve*Ce)/Vb

Cv = ((Qh*Ch)/Php+(Qm*Cm)/Pmp+(Qo*Co)/Pop)/Qtot

Cvb = (Vp*Cv+Ve*Ce)/Vb

# Display graph

# C　: umol/L = X 27 ug/L = X 0.027 ug/mL

plt.plot(sol.t, Cb*0.027, label="Cb")

plt.plot(sol.t, Cvb*0.027, label="Cvb")

plt.legend()

plt.show()

**Supplemental material 2 Conceptual image of the method for estimating inhaled hydrogen cyanide gas concentration and time**


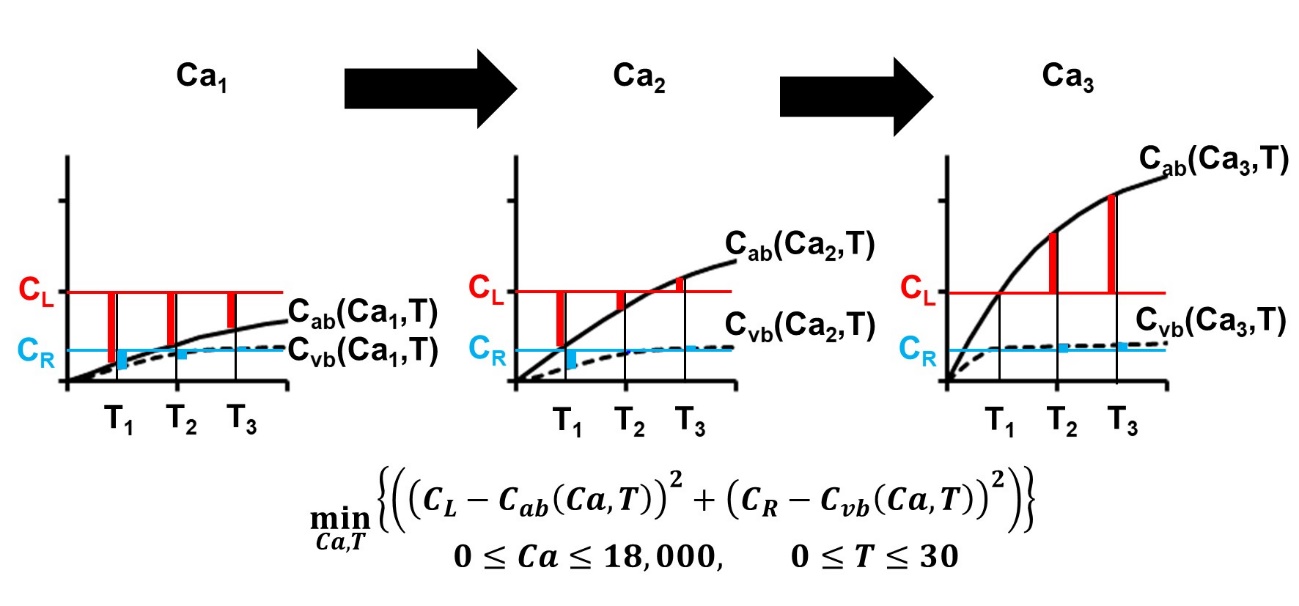


**Supplemental material 3 Python script for estimating inhaled hydrogen cyanide gas concentration and time**

Note: By entering the measured cyanide concentrations in the left and right cardiac blood after “Crh=” and “Clh=” in the following script, respectively, and executing it, the estimated inhaled hydrogen cyanide gas concentration and time can be obtained.

# -*- coding: utf-8 -*-

# preparation of numpy and plot

import numpy as np

from scipy.integrate import solve_ivp

Ca0 = []

Del_min = []

Del_min_time = []

for value in [i*0.5 for i in range(0,751)] :

# definition of parameters

Ppa = 281 # Ppa: Partition coefficients of plasma/air

Php = 5.1 # Php: Partition coefficients of liver/plasma

Pmp = 2.8 # Pmp: Partition coefficients of muscle/plasma

Pop = 5.4 # Pop: Partition coefficients of other tissues/plasma

Qalv = 16.5 # Qalv: Alveolar ventilation

Qtot = 10.7 # Qtot: Cardiac output

Qh = 1.6 # Qh: Liver blood flow

Qm = 4.9 # Qm: Muscle blood flow

Qo = 4.2 # Qo: Other tissues blood flow

Fp = 0.6 # Fp: Plasma fraction in whole blood

Cle = 65.9 # Cle: Clearance flow between blood and erythrocytes

Vp = 2.9 # Vp: Compartment volume of plasma

Ve = 1.9 # Ve: Compartment volume of erythrocytes

Vh = 1.6 # Vh: Compartment volume of liver

Vm = 35 # Vm: Compartment volume of muscle

Vo = 18 # Vo: Compartment volume of other tissues

Vb = 4.8 # Vb: Compartment volume in whole blood

Emax = 140 # Emax: Maximum binding to erythrocytes

Kaff = 1 # Kaff: Affinity constant to erythrocytes from plasma

Kscn = 0.01 #Kscn : second-order rate constant for thiocyanate formation

Kfs = 2.2 # Kfs: zero-order formation rate of sulfur donors

Kes = 0.0027 # Kes: first-order elimination rate constant

Clr = 0.0041 # Clr: first-order metabolic clearance of CN via remaining pathways

Ca = value # CN conc. in air (umol/L), 1 umol/L = 0.027 ug/mL = 24 ppm, 270 ppm = 0.30 ug/mL = 11 umol/L

Ca0.append(Ca)

# definition of variables

# C = [0 Cp, 1 Ce, 2 Ch, 3 S, 4 Cm, 5 Co]

init = [0, 0, 0, 1, 0, 0]

#Cp: CN conc. in plasma

#Ce: CN conc. in erythrocytes

#Ch: CN conc. in liver

#S: sulfur donors conc.

#Cm: CN conc. in muscle

#Co: CN conc. in other tissues

# Setting of time span of simulation

t_span = [0.0, 30.0]

dt = 0.05

t_eval = np.arange(*t_span, dt)

# Definition of ordinary differential equation and initial values

def func(t,C):

Cp, Ce, Ch, S, Cm, Co = C

dCp = (Qalv*(Ca-(Cp/Ppa))+Cle*(Ce/(Emax/(Cp+Kaff))-Cp)+Fp*Qtot*(((Qh*Ch)/Php+(Qm*Cm)/Pmp+(Qo*Co)/Pop)/Qtot-Cp))/Vp

dCe = Cle*(Cp-(Ce/(Emax/(Cp+Kaff))))/Ve

dCh = (Fp*Qh*(Cp-(Ch/Php))-Kscn*Ch*S-(Kfs-Kscn*Ch*S-Kes*S))/Vh

dS = Kfs-Kscn*Ch*S-Kes*S

dCm = Fp*Qm*(Cp-Cm/Pmp)/Vm

dCo = Fp*Qo*(Cp-Co/Pop)/Vo

return np.array([dCp, dCe, dCh, dS, dCm, dCo])

sol = solve_ivp(func, t_span, init, method='RK45', t_eval=t_eval)

Cp, Ce, Ch, S, Cm, Co = sol.y

#Metscn: CN conc. metabolized via the SCN route

#Cb: CN conc. in whole blood

#Cv: CN conc. in vein (mixed venous plasma)

#Cvb: CN conc. in whole blood in vein

Metscn = Kscn*Ch*S

Cb = (Vp*Cp+Ve*Ce)/Vb

Cv = ((Qh*Ch)/Php+(Qm*Cm)/Pmp+(Qo*Co)/Pop)/Qtot

Cvb = (Vp*Cv+Ve*Ce)/Vb

#print(Cb)

#print(Cvb)

#Measured blood CN value (input)

Crh =

Clh =

Del = np.empty(len(sol.y[0]))

Del = (Crh - Cvb*0.027)**2 + (Clh - Cb*0.027)**2

#Form list minimum sum of squares of differences, and the time on getting it

Del_min.append(min(Del))

Del_min_time.append(1/20 * np.argmin(Del))

print(min(Del))

Del_min_index = Del_min.index(min(Del_min))

#Estimated Ca on getting minimum sum of squares of differences

print(Ca0[Del_min_index])

print(24*Ca0[Del_min_index])

#Estimated HCN inhalation time on getting minimum sum of squares of differences

print(Del_min_time[Del_min_index])

#Minimum sum of squares of differences

print("Del =", min(Del_min))

**Supplemental material 4 Sensitivity analysis results for inhaled hydrogen cyanide gas concentration and time**

| No. | CN conc. (μg/mL) | | Estimated inhaled HCN gas conc. | Estimated inhalation time |
| --- | --- | --- | --- | --- |
|  | R | L | (ppm) | (min) |
| 17 | 0.408 | 2.295 | 11,988 | 0.05 |
|  | 0.408 | 2.700 | 14,112 | 0.05 |
|  | 0.408 | 3.105 | 16,260 | 0.05 |
|  | 0.480 | 2.295 | 12,072 | 0.05 |
|  | 0.480 | 2.700 | 14,280 | 0.05 |
|  | 0.480 | 3.105 | 16,632 | 0.05 |
|  | 0.552 | 2.295 | 12,168 | 0.05 |
|  | 0.552 | 2.700 | 14,268 | 0.05 |
|  | 0.552 | 3.105 | 16,404 | 0.05 |
| 10 | 1.950 | 3.400 | 852 | 9.45 |
|  | 1.950 | 4.000 | 1,200 | 6.70 |
|  | 1.950 | 4.600 | 1,560 | 5.10 |
|  | 2.300 | 3.400 | 648 | 22.10 |
|  | 2.300 | 4.000 | 996 | 13.95 |
|  | 2.300 | 4.600 | 1,356 | 10.05 |
|  | 2.645 | 3.400 | 588 | 29.90 |
|  | 2.645 | 4.000 | 804 | 25.10 |
|  | 2.645 | 4.600 | 1,152 | 17.10 |
| 7 | 1.020 | 1.445 | 1,392 | 0.30 |
|  | 1.020 | 1.700 | 2,424 | 0.20 |
|  | 1.020 | 1.955 | 3,660 | 0.15 |
|  | 1.200 | 1.445 | 468 | 0.95 |
|  | 1.200 | 1.700 | 1,260 | 0.40 |
|  | 1.200 | 1.955 | 2,268 | 0.25 |
|  | 1.380 | 1.445 | 48 | 26.80 |
|  | 1.380 | 1.700 | 336 | 1.70 |
|  | 1.380 | 1.955 | 876 | 0.70 |
